# A‐Site Cation Replacement of Hydrazinium Lead Iodide Perovskites by Borane Ammonium Ions: A DFT Calculation

**DOI:** 10.1002/open.202300207

**Published:** 2023-12-04

**Authors:** Mohammad Tanvir Ahmed, Shariful Islam, Farid Ahmed

**Affiliations:** ^1^ Department of Physics Jashore University of Science and Technology Jashsore Bangladesh; ^2^ Department of Physics Jahangirnagar University Dhaka Bangladesh

**Keywords:** DFT, perovskite, DOS, band gap, tolerance factor

## Abstract

Organometallic perovskites have become one of the most common multifunctional materials in optoelectronic research fields. This research studies density functional theory calculation on orthorhombic hydrazinium lead iodide (N_2_H_5_PbI_3_) perovskite by replacing A‐site cation with a borane ammonium (BH_2_NH_3_^+^) ion. The perovskite showed a significant structural deformation and an orthorhombic to triclinic phase transition due to A‐site ion replacement. The N_2_H_5_PbI_3_ perovskite has a band gap of 1.64 eV, suitable for the solar cell absorber layer. The band gap has increased to 2.12 eV after complete A‐site ion replacement. All structures showed a high absorption coefficient over 10^4^ cm^−1^ in the low wavelength region and an increase in refractive index from 2.5 to 2.75 due to ion replacement. All the structures showed high optical conductivity of 10^15^ s^−1^ order in the blue wavelength region. These new perovskite structures hold the potential to provide a revolution in optoelectronic research.

## Graphical abstract

The NH_2_NH_3_^+^ A‐site cations of N_2_H_5_PbI_3_ perovskite have been replaced by BH_2_NH_3_^+^ iond to study the structural and optoelectronic characteristics of the designed perovskite. A significant change in crystal structure along with an increase in band gap is observed due to BH_2_NH_3_^+^ addition. All the structures show a good optical response suitable for various optoelectronic research.

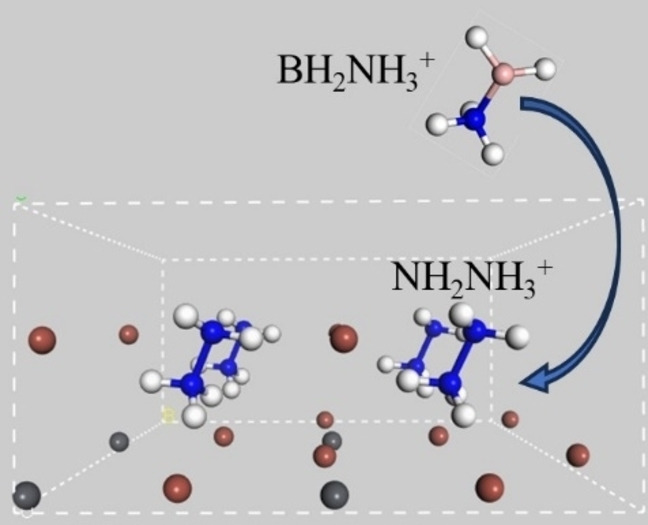

## Introduction

The typical formula for hybrid organic‐inorganic perovskites (OIPs) is AMX_3_, where A, M, and X represent monovalent organic cation, divalent metal cation, and halogen ion, respectively.[^1^, ^2^] These materials are multifunctional and have a broad range of uses, including in photodetectors, solar cells (SCs), field‐effect transistors (FET), light‐emitting diodes, lasers, light‐emitting electrochemical cells, and more.[^3^, ^4^, ^5^, ^6^] According to the National Renewable Energy Laboratory report, one of the best applications of OIPs in SC technology is a remarkable light absorber with a maximum power conversion efficiency (PCE) of 25.5 %.^[7]^

The most serious drawback of OIPs is their lack of stability in the presence of oxygen, moisture, high temperatures, electric fields, light, and light‐like substances. Compositional engineering of the perovskite structure may help to alleviate this issue partly. Specific arrangements of the A, M, and X ions can only create the complicated AMX_3_ halide perovskite structure. A criterion, tolerance factor (t), was proposed by Goldschmidt (1920) that allows well understanding of which mixture of atomic species is suitable to produce a stable perovskite structure. The tolerance factor can be calculated from Equation 1,^[8]^

(1) $$t=\frac{R_{x}+R_{A}}{\sqrt{2}\;\left(R_{x}+R_{M}\right)}\;$$

where R_A_, R_M,_ and R_X_ represent the ionic radii of A, M, and X‐site atoms, respectively. A value of t≈1 denotes a successful perovskite creation with a great fit whereas, if “t” does not lie within the range 0.81–1.11, MX_6_ octahedra with lower dimensional connections, such as 2D layers, 1D chains, or 0D MX6 octahedral clusters, would develop instead of the 3D perovskite structure.^[8]^ The most commonly researched OIP is CH_3_NH_3_PbI_3_ (MAPI) containing methylammonium (CH_3_NH_3_^+^) ion in the A site of ionic radius 2.17 Å resulting in a tolerance factor of 0.91.^[8]^ On the other hand, Hydrazinium ion (HA^+^), a candidate for A site cation, has a similar ionic radius to that of methylammonium (MA) with a different geometry since the NH_2_ group in HA^+^ replaces the CH_3_ group in MA^+^.^[9]^ The hydrazinium lead iodide (HAPI) also holds a tolerance factor of 0.912.

Very few researches on HAPI have been reported to the present. According to the theoretical prediction, HAPI is a stable structure and alternative to MAPI perovskite in optoelectronic (OE) research.^[10]^ Akbulatov et al., in 2016, experimentally synthesized MA_1‐x_HA_x_PbI_3_ perovskites for the solar cell absorber layer, where maximum efficiency up to 11.6 % was observed for the MA_0.9_HA_0.1_PbI_3_ perovskites with an increase in stability.^[9]^ The synthesized HAPI showed a hexagonal symmetry. In 2018, Campbell et al. studied hexagonal HAPI both theoretically and experimentally and obtained a 2.48–2.70 eV band gap which is comparatively high for solar cell active material.^[11]^ However, the observed properties make HAPI a potential material for numerous optoelectronic research. They also suggested the possibility of a cubic HAPI structure. Tsarev et al., in 2018, experimentally replaced MA^+^ ion partially with HA^+^ ion in the CH_3_NH_3_SnI_3_ perovskite and reported that adding HA^+^ improved the device efficiency via decreasing void density in the morphology.^[12]^ Li et al., in 2019, reported a cation displacement technique to synthesize CH_3_NH_3_SnI_3_ from N_2_H_5_SnI_3_ perovskite, which showed a reduction of Sn^4+^ and a performance enhancement with 7.13 % PCE.^[13]^ The orthorhombic phase of HAPI was studied theoretically, which showed a band gap of 1.6 eV and a high absorption coefficient over 10^4^ cm^−1^, suitable for the solar cell‘s active layer.^[14]^ Although various studies on HAPI have been reported over the last few years, the effect of ion replacement can be a new pathway for modifying various properties, which requires further studies. Enhancement of optical absorption and tuning of band gap can be obtained through ion replacement which may reveal potential materials for OE research.

Here, we modelled and studied orthorhombic HAPI perovskite and replaced the HA^+^ ion with BH_2_NH_3_^+^ (Borane ammonium, BA^+^) ion. BA^+^ ion can be a potential candidate for A‐site perovskite cation which has not been studied previously. We have observed the variation of geometrical, optical, and electronic characteristics of the designed perovskites due to ion replacements. To our knowledge, the geometry and optoelectronic properties of BH_2_NH_3_PbI_3_ (BAPI) perovskite have never been reported. This study showed that HA_1‐x_BA_x_PbI_3_ perovskites are an alternative candidate for MAPI in various OE research.

## Computational Methods

The Cambridge Serial Total Energy Package (CASTEP) was used to perform density functional theory (DFT) calculation using the plane‐wave ultrasoft pseudopotential.^[15]^ The contribution to OE behavior was studied by considering lead (Pb) and iodine (I) valance electrons. For the exchange‐correlation correction, the generalized gradient approximation of the Perdew–Burke–Ernzerhof method was utilized.^[16]^ Geometry optimization was performed using the Broyden–Fletcher–Goldfarb–Shanno (BFGS) minimization algorithm.^[17]^ For OE properties, we construct 2×2×1 supercells of each crystal perovskite structure where a 3×3×3 k‐point mesh following the scheme of Monkhorst–Pack, which was employed to compute the electronic and optical properties.^[15]^ Throughout the computation, a plane wave cutoff energy of 500 eV was used. The geometries of the structures were optimized using convergence criteria of pressure to be 0.05 GPa, 3×10^−2^ eV/atom for maximum force, 1.0×10^−5^ eV/atom for total energy, and 0.001 Å for displacement.^[15]^

## Results and Discussion

### Optimized Geometry

We have modelled five mix cation perovskite with the general formula HA_(1‐x)_BA_x_PbI_3_ (x=0, 0.25, 0.5, 0.75, 1). For simplicity, we defined the perovskites HAPbI_3_, HA_0.75_BA_0.25_PbI_3_, HA_0.5_BA_0.5_PbI_3_, HA_0.25_BA_0.75_PbI_3_, and BAPbI_3_, by HBPI0, HBPI25, HBPI50, HBPI75, and HBPI100, respectively.

Table 1 shows the geometrical variation of perovskite crystals due to the replacements of the A‐site cation. The lattice parameters of the unit cell structures are obtained from their supercell geometry analysis. According to the study, the HBPI0 possessed an orthorhombic phase which satisfies the previous report.^[14]^ The lattice parameters of HBPI0 are quite analogous to the ones of MAPI perovskite.[^18^, ^19^]

**Table 1:** Lattice parameters of the modelled perovskite structures.

Material	Lattice parameters					
	a (Å)	b (Å)	c (Å)	α (°)	β (°)	γ (°)
HBPI0	6.502	6.451	6.364	~90	~90	~90
HBPI25	6.493	6.449	6.439	88.8	90.4	91.9
HBPI50	6.446	6.449	6.492	88.9	90.7	92.5
HBPI75	6.315	6.362	7.138	88.4	89.8	91.2
HBPI100	6.309	6.219	7.189	93.9	88.9	91.9

**Figure 1:**
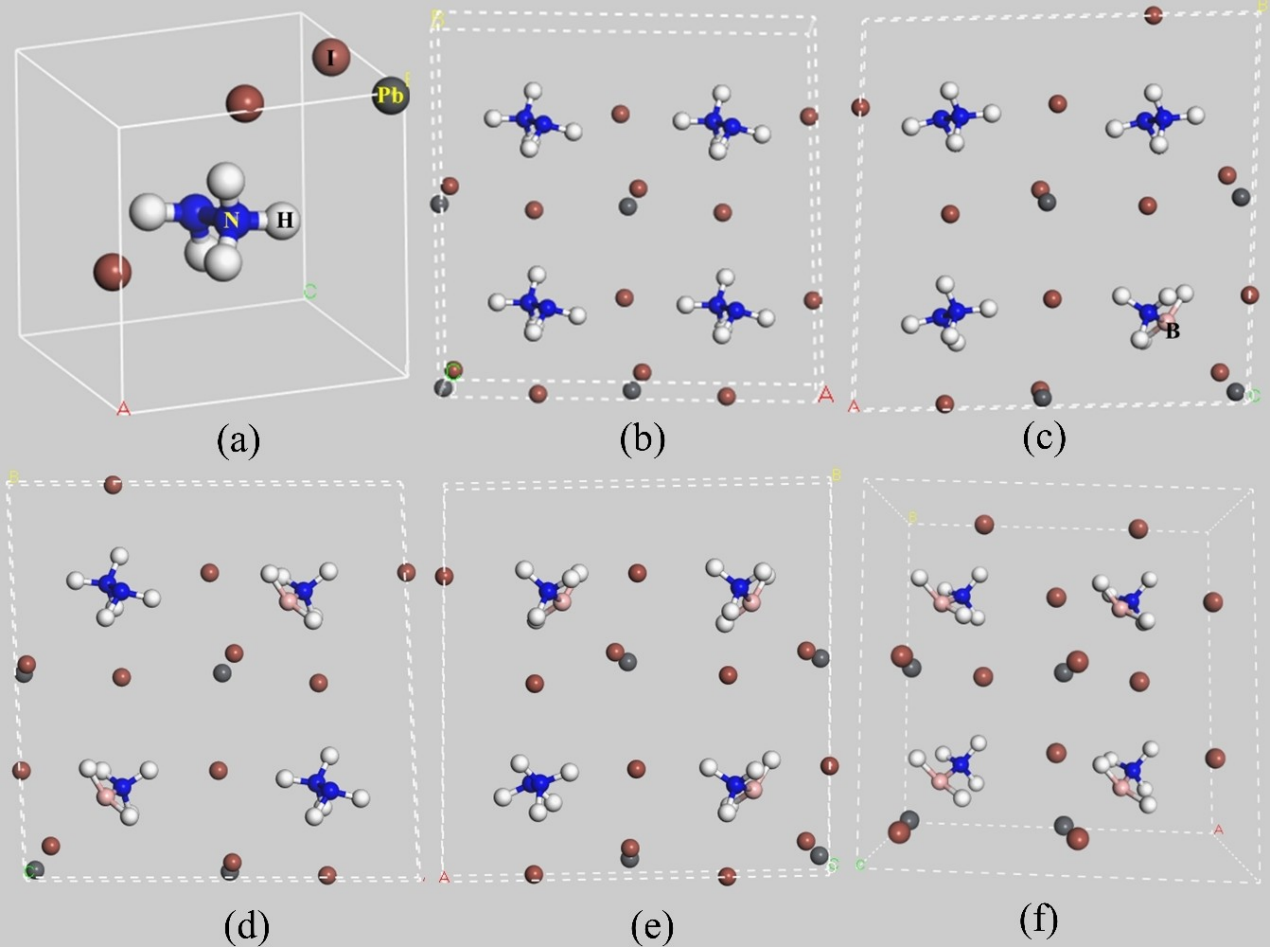
Optimized geometry of a) HAPI unit cell, b) HBPI0, c) HBPI25, d) HBPI50, e) HBPI75, f) HBPI100.

It appears that by replacing HA^+^ ion with BA^+^ ion, the structure tends to deform towards the triclinic phase. Due to the length and width variation of BA^+^ from HA^+^, the lattice parameter of the perovskites decreases in the x and y directions with increasing BA^+^ concentration, whereas it increases in the z‐direction. Since the effective ionic radius of BA^+^ has not been reported before, we have calculated the radius to be about 2.48 Å from the optimized structure through the previously reported method.^[20]^ Hence BAPI possessed a tolerance factor of 0.967. Hence the BA^+^ will fit in the cavity of the PbI_6_ octahedral framework.^[8]^ Hence, all the studied structures can show fine structural stability, and based on the tolerance factor, the structural stability can be compared as follows: HBPI100>HBPI75>HBPI50>HBPI25>HBPI0. The geometries of the optimized perovskite structures are demonstrated in Figure 1.

### Charge Distribution

Both Mulliken and Hirshfield's charges of HBPI0 are analogous to previous research on orthorhombic HAPI.^[14]^

Table 2 and Table 3 show the Mulliken and Hirshfield charge distribution among the perovskite crystal elements. N and I atoms show a partial negative charge in every structure due to their high electronegativity. In contrast, Pb and H atoms possess partial positive charge, that is, bonding electrons are displaced from Pb and H atoms towards I and N atoms, respectively. Since Boron is less electronegative than Nitrogen, it possesses a slightly negative charge less than Nitrogen. Both Mulliken and Hirshfield's charges satisfy the explanation. Both Mulliken and Hirshfield's charges of HBPI0 are analogous to previous research on orthorhombic HAPI.^[14]^

**Table 2:** Mulliken charges of the elements.

Material	N	B	Pb	I	H
HBPI0	−0.66	–	0.74	−0.44	0.38
HBPI25	−0.684	−0.16	0.715	−0.383	0.335
HBPI50	−0.732	−0.16	0.743	−0.353	0.297
HBPI75	−0.798	−0.18	0.77	−0.318	0.264
HBPI100	−0.9	−0.19	0.88	−0.31	0.226

**Table 3:** Hirshfield charges of the elements.

Material	N	B	Pb	I	H
HBPI0	−0.065	–	0.26	−0.22	0.102
HBPI25	−0.077	−0.01	0.29	−0.18	0.08
HBPI50	−0.08	−0.01	0.30	−0.16	0.06
HBPI75	−0.088	−0.01	0.32	−0.148	0.046
HBPI100	−0.09	−0.01	0.34	−0.13	0.032

**Figure 2:**
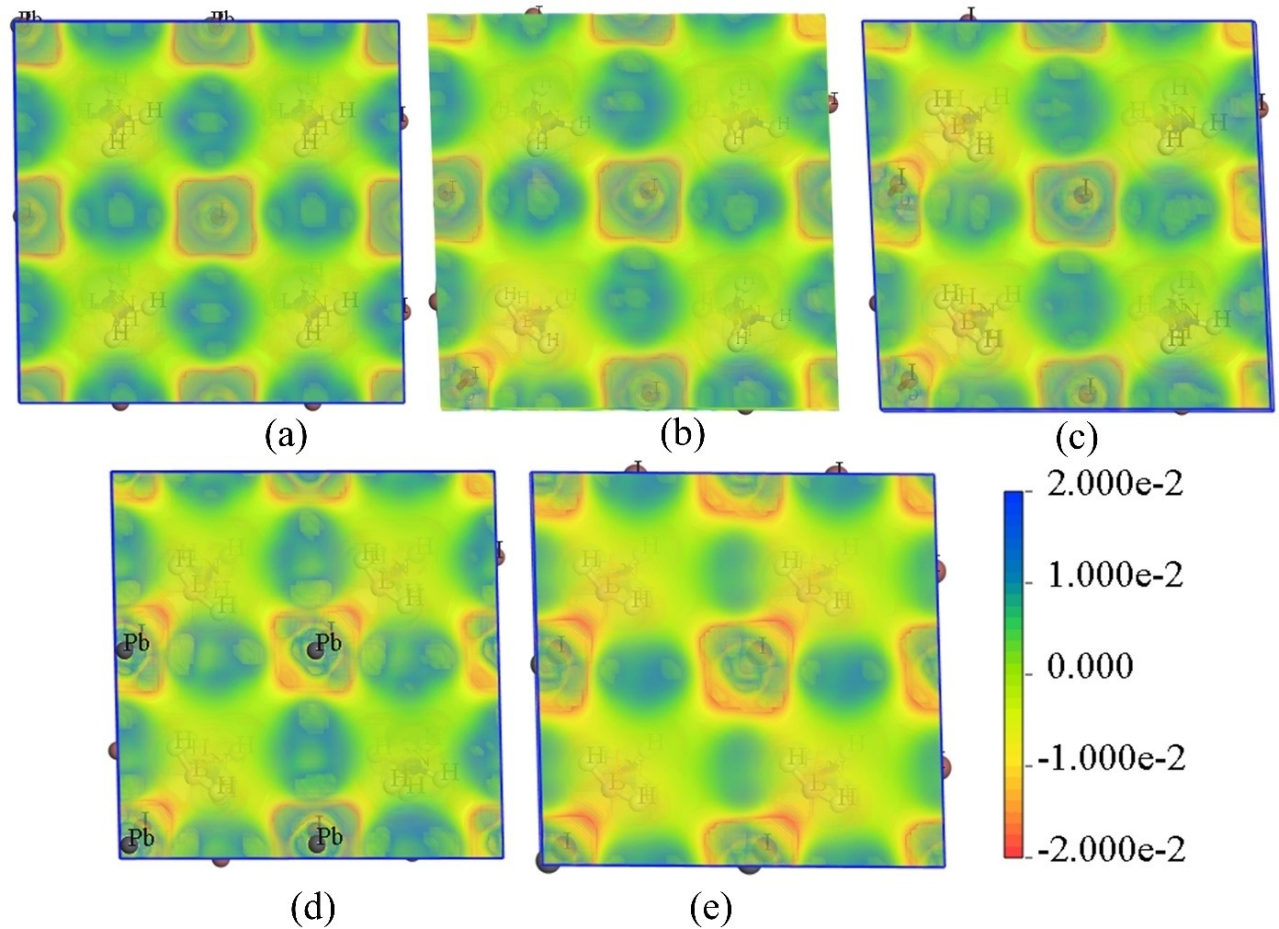
Electron density map of a) HBPI0, b) HBPI25, c) HBPI50, d) HBPI75, e) HBPI100 crystal surface.

Figure 2 shows the electrostatic potential map of the perovskite structures. The red region represents a high electron‐dense region, whereas the blue color represents the electron deficit region. The Pb ions show comparatively positive potential, whereas I ions in the background are negative. After the introduction of BA^+^ ions, the B ion site shows a slight color transformation from light green to yellow, representing an increase in negative potential in the region.

### Band Structure

**Figure 3:**
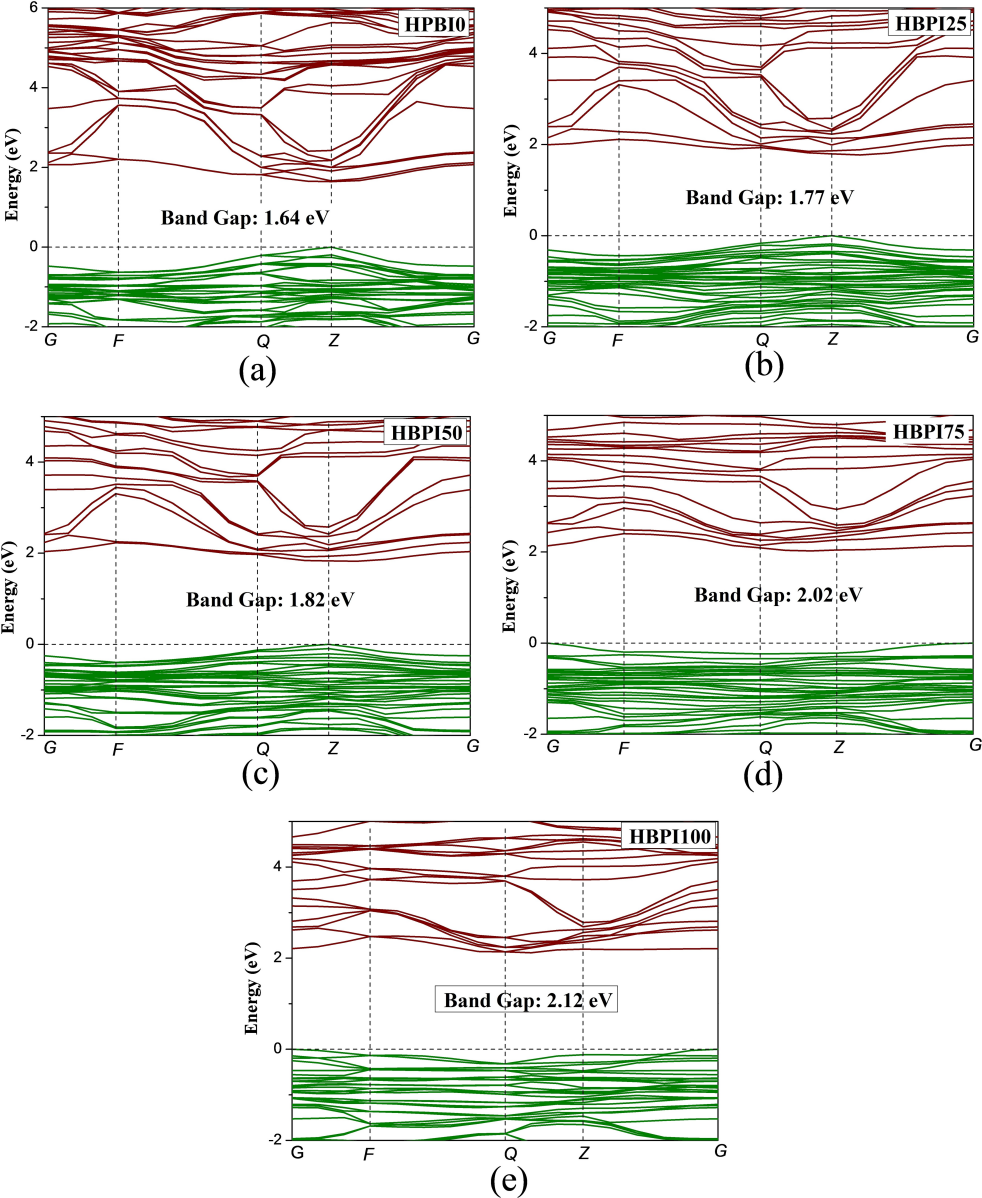
Band structure of a) HBPI0, b) HBPI25, c) HBPI50, d) HBPI75, e) HBPI100 crystals.

Figure 3 shows the band structure of the perovskite crystals. The band structures have been calculated along the Brillouin zone symmetry points *G→F→Q→Z→G*. The HBPI0, HBPI25, and HBPI50 showed a direct band gap in the ***Z*** k‐point. In contrast, the band gap shifted to ***G*** k‐point for HBPI75 and HBPI100 perovskites. The HBPI0 has a band gap of 1.64 eV, which matches with the previous study and is close to the band gap of MAPI perovskites,^[14]^ making HBPI0 a potential alternative OE material for MAPbI_3_ perovskites in various applications like solar cells, LASER, LED, etc. The orthorhombic HBPI0 has a significantly less band gap than the hexagonal HBPI0.^[11]^ After replacing HA^+^ with BA^+^ ions, the band gap increased significantly. There is a significant impact of A‐site cation on the band gap of perovskites.^[21]^ Hence, the variation of the band gap is observed due to A‐site ion replacement. The variation of band gap can be caused due to structural deformation after HA^+^ replacements. Since HBPI100 shows a more deformed structure than HBPI0, HBPI100 has the maximum band gap. The band gap of HBPI0, HBPI25, HBPI50, HBPI75, and HBPI100 are 1.64 eV, 1.77 eV, 1.82 eV, 2.02 eV, and 2.12 eV, respectively. The band gap of HBPI100 is analogous to the one of MAPbBr_3_ perovskite.[^22^, ^23^] Hence a significant tuning of the band gap is possible via A‐site cation replacement which can be beneficial for various OE applications.

#### Density of States (DOS)

**Figure 4:**
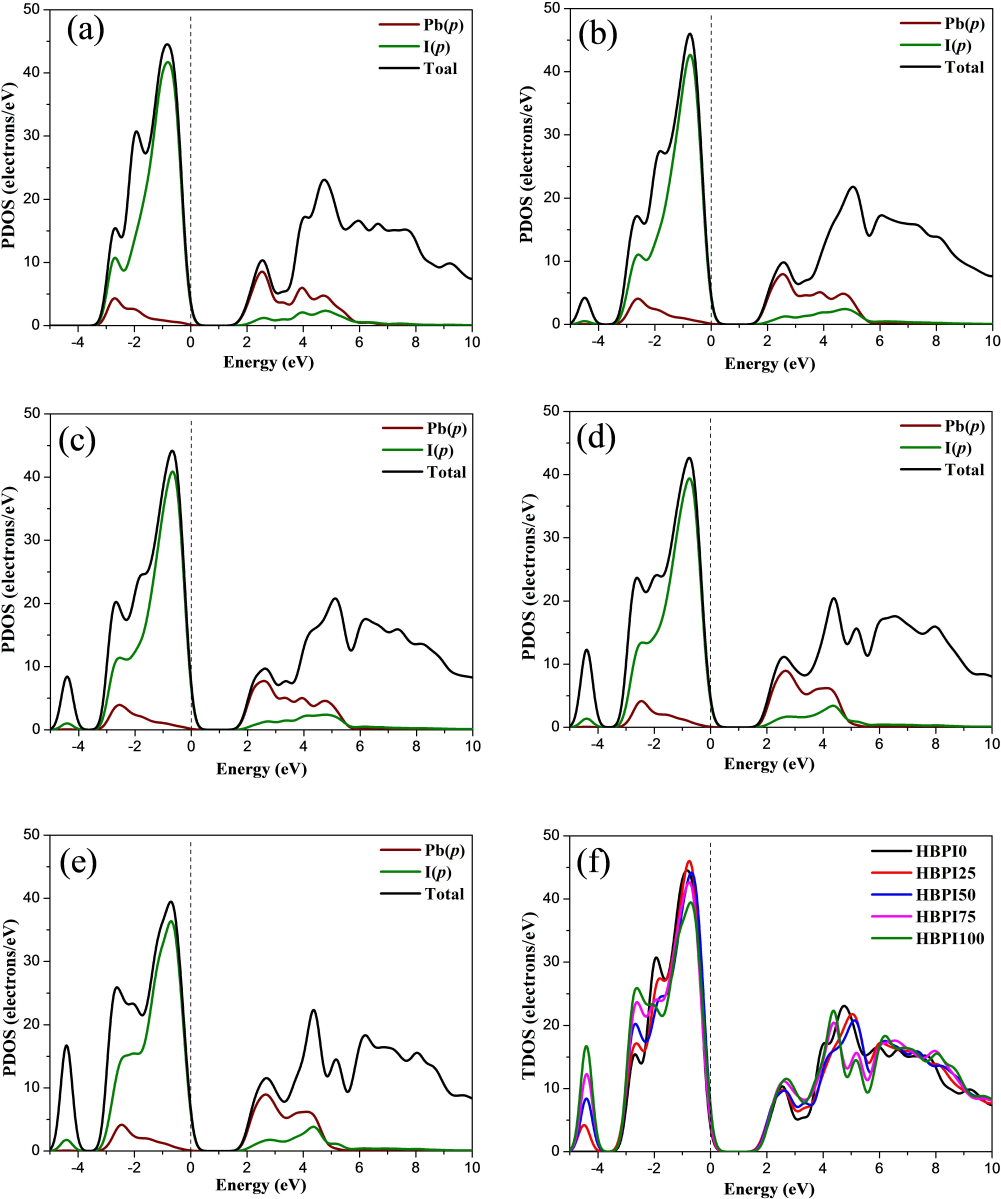
PDOS spectra of a) HBPI0, b) HBPI25, c) HBPI50, d) HBPI75, e) HBPI100, and f) total DOS of all structure.

Figure 4 shows the partial DOS (PDOS) and Total spectra of the designed perovskite structures. The 5*p* orbital of Iodine contributed to the valance band maximum (VBM), as shown in these PDOS figures, while the conduction band minimum (CBM) is composed of Pb‐6*p* orbitals.^[24]^ The electronic configuration of Pb(II) is 6 *s* ^2^6*p* ^0^ whereas for halides, it is n*p* ^6^ (n=2,3,4); hence the halide *p* orbitals contribute the most to the VBM and CBM. Since the electrical band edge is not affected directly by the A‐site cations (i. e., HA^+^, BA^+^), the contribution to VBM and CBM remains almost identical for all the perovskites.^[21]^

### Optical Properties

The dielectric function (*ϵ*) is one of the vital properties for understanding the optical properties, which depends on the frequency (ω ) of the incident photon. The real, imaginary part of the dielectric function (ϵ_r_, ϵ_i_) and the complex *ϵ* can be calculated from the following equations (2), (3) and 4.[^25^, ^26^, ^27^]

(2) $$\epsilon_{r}\left(\omega \right)=1+\frac{2}{\pi }P\int_{0}^{\infty }\frac{\omega^{{}^{'}\epsilon_{i}\left(\omega^{'}\right)}d\omega {}^{'}}{\omega^{{}^{'}2}-\omega^{2}}$$

(3) $$\epsilon_{i}\left(\omega \right)=\left(\frac{4\pi^{2}e^{2}}{m^{2}\omega^{2}}\right)\sum_{x,y}\int x\left|M\right|y^{2}f_{x}\left(1-f_{x}\right)\delta \left(E_{f}-E_{x}-\omega \right)d^{3}K$$

(4) $$\epsilon \left(\omega \right)=\epsilon_{r}\left(\omega \right)+i\epsilon_{i}\left(\omega \right)$$

where, *P*, *M*, *x*, and *y* represent the principle value of integral, dipole matrix, initial state and final state respectively. *f_x_* is the fermi function and *E_x_* is electron energy in the *x* ^th^ state. The real and imaginary refractive index (*n*, *k*) is related to the dielectric function by the following equations (5) and 6.[^28^, ^29^]

(5) $$\epsilon_{r}=n^{2}-k^{2}$$

(6) $$\epsilon_{i}=2nk$$

The absorption coefficient (α ), reflectivity (*R*), conductivity (σ ), and loss function (φ ) can be obtained from the following equations (7–10.[^18^, ^30^, ^31^]

(7) $$\alpha \left(\omega \right)=\frac{\sqrt{2}\omega }{c}{\left[\sqrt{\epsilon_{r}^{2}\left(\omega \right)+\epsilon_{i}^{2}\left(\omega \right)}-\epsilon_{r}\left(\omega \right)\right]}^{\frac{1}{2}}$$

(8) $$R\left(\omega \right)=\frac{{\left(1-n\right)}^{2}+k^{2}}{{\left(1+n\right)}^{2}+k^{2}}$$

(9) $$\sigma =\frac{\alpha nc}{4\pi }$$

(10) $$\varphi =\frac{\epsilon_{i}\left(\omega \right)}{\epsilon_{r}^{2}\left(\omega \right)+\epsilon_{i}^{2}\left(\omega \right)}$$

where, *c* is the speed of light.

**Figure 5:**
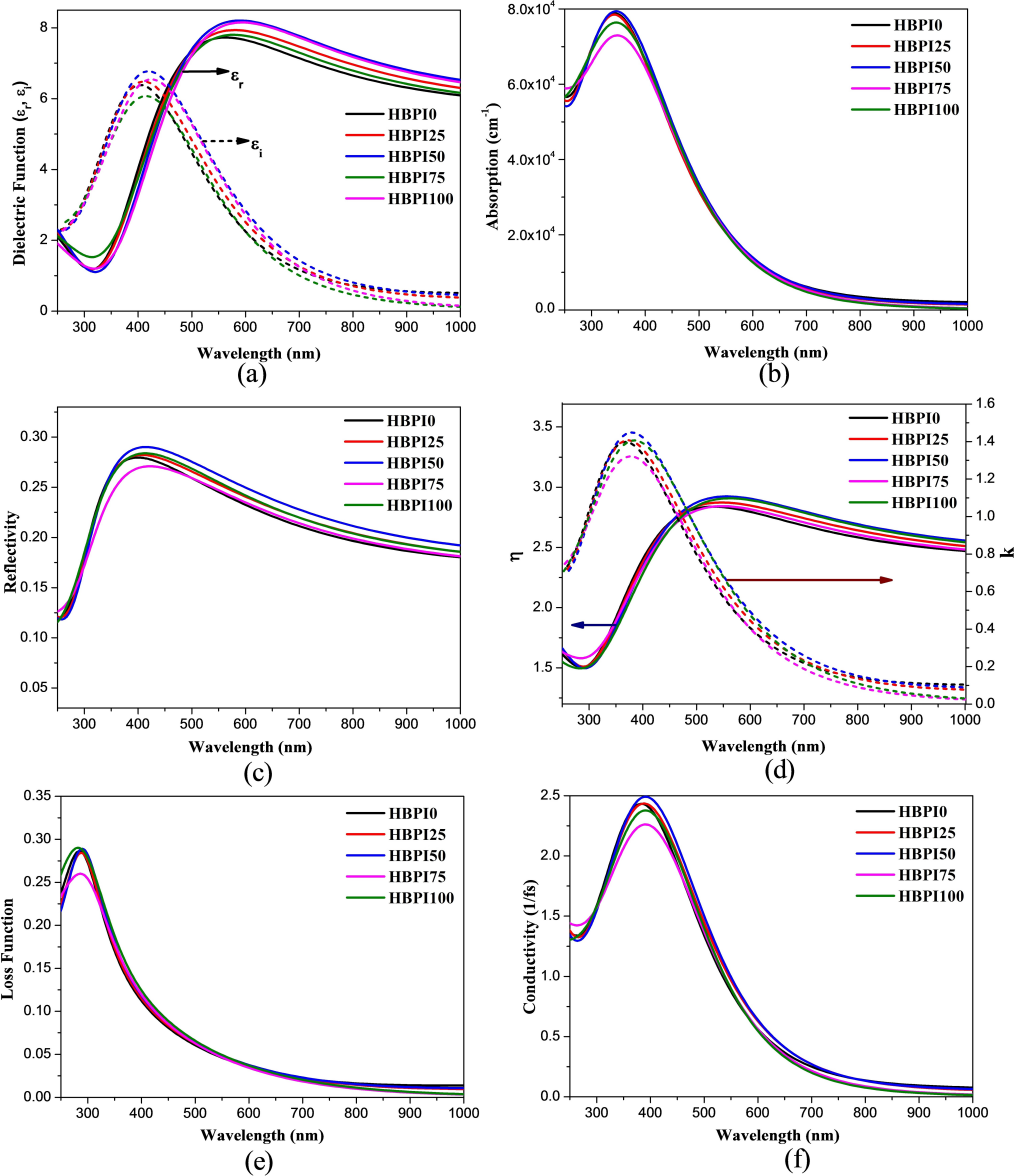
(a) Dielectric function, (b) absorption coefficient, (c) reflectivity, (d) refractive index, (e) loss function, and (f) optical conductivity.

Figure 5(a) shows the real and imaginary parts of the dielectric function. The real part (ϵ_r_) indicates polarization, whereas the imaginary part (ϵ_i_) represents energy dissipation, that is, absorption of light inside the material.[^32^, ^33^] The imaginary part of the dielectric function of HBPI0 shows a strong peak near 402 nm which suffers a slight variation due to the replacement of HA^+^ addition of BA^+^. The real part shows a significant impact in the whole visible spectrum region. The maximum values of ϵ_r_ of the structures are observed around 550–580 nm which further decreased with increasing wavelength in a similar manner to MAPI.^[34]^

Figure 5(b, c) shows the optical absorption coefficient and reflectivity of the designed perovskites. The absorption coefficients of all the structures are very high (over 10^4^ cm^−1^) in the shorter wavelength region, making them ideal for numerous OE applications. However, the absorption coefficient is comparatively less than both theoretically and experimentally observed MAPI perovskites.[^35^, ^36^, ^37^] The absorbance nature of HBPI0 is analogous to previous research.[^9^, ^14^] Reflectivity is a measure of the fraction of reflected energy from the surface of crystals; hence is an essential property for OE applications.^[38]^ A material with less reflectivity can provide better OE performance due to less energy loss. All the structures showed 20 % to 30 % reflection of incident energy in the visible wavelength region. The reflectivity has increased very slightly due to BA^+^ incorporation.

The refractive index (η) of the perovskites is about 2.5–2.75 in the visible wavelength region Figure 5(d), which is slightly higher compared to MAPI perovskites,^[39]^ which signifies that HBPI will cause more reflection of incident light than MAPI. The refractive index has increased slightly after the replacement of HA^+^ ions. The extinction coefficient (k) represents the absorption in the UV region of the electromagnetic spectrum. It is a measure of the fraction of absorbed light per unit distance and is related to the imaginary part of the complex refractive index.^[40]^ The extinction coefficient is quite analogous to the experimental result of MAPI, with a slightly lower value in the visible region, signifying comparatively less adsorption of visible light than MAPI.^[41]^ The extinction coefficient of all the perovskite structures showed a strong maximum in the lower wavelength region of the visible spectrum.

Plasma oscillations are generated through the interaction of light with the free electrons of the lattice. These oscillations are responsible for loss function peaks [Figure 5(e)], that is, plasmon peaks.^[42]^ The plasmon peaks for perovskites are observed in the near UV region, and the peak positions are slightly blue‐shifted after BA^+^ incorporation. Optical conductivity represents the rise in electrical conductivity due to the absorption of photons [Figure 5(f)]. The maximum conductivity is observed in the blue wavelength region due to higher energy. The conductivity decreases for all perovskites with decreasing the incident photon energy. The peak shifting of conductivity after BA^+^ incorporation is minimal, and the maximum conductivity value is observed for the HBPI50 perovskite.

## Conclusions

The structural, electronic, and optical properties and their variation due to the replacements of A site ion of N_2_H_5_PbI_3_ perovskite have been studied by DFT calculations. All the designed perovskites show fine structural stability due to the suitable range of tolerance factors. The N_2_H_5_PbI_3_ perovskite showed a structural deformation and a phase transition from orthorhombic to triclinic due to the replacement of N_2_H_5_^+^ ion by BH_2_NH_3_^+^ ion. The surface charge distribution has slightly varied with an increase of band gap from 1.64 eV to 2.12 eV after the complete replacement of the A site cation. All the structures have a strong absorption coefficient (over 10^−4^ cm^−1^) for the low wavelengths of the visible spectrum with a maximum 30 % reflection of incident energy. The optical conductivity showed peak value in the blue wavelength region, suggesting the conductivity can rise greatly by illumination of blue light. The replacement of A‐site cations can be a possible way of band gap tuning for different OE applications. The electronic bandgap in the visible energy range, high absorption coefficient, and conductivity suggest that the studied structures hold significant potential in numerous OE applications.

## Author Contributions

All authors contributed to the study‘s conception and design. Material design, data collection, analysis, and draft writing were performed by Mohammad Tanvir Ahmed. Shariful Islam performed the revision and editing of the original draft. Farid Ahmed supervised the whole research. All authors read and approved the final manuscript.

## Conflict of interest

The authors have no conflict of interest.

The authors have no relevant financial or non‐financial interests to disclose.

1

## Supporting information

As a service to our authors and readers, this journal provides supporting information supplied by the authors. Such materials are peer reviewed and may be re‐organized for online delivery, but are not copy‐edited or typeset. Technical support issues arising from supporting information (other than missing files) should be addressed to the authors.

Supporting Information

## Data Availability

Research data has been shared as supplementary information.
